# The relationship between neuroticism, major depressive disorder and comorbid disorders in Chinese women

**DOI:** 10.1016/j.jad.2011.06.053

**Published:** 2011-12

**Authors:** Jing Xia, Qiang He, Yihan Li, Dong Xie, Suoyu Zhu, Jing Chen, Yuan Shen, Ning Zhang, Yan Wei, Chunfeng Chen, Jianhua Shen, Yan Zhang, Chengge Gao, Youhui Li, Jihong Ding, Wenwu Shen, Qian Wang, Meiyue Cao, Tiebang Liu, Jinbei Zhang, Huijun Duan, Cheng Bao, Ping Ma, Cong Zhou, Yanfang Luo, Fengzhi Zhang, Ying Liu, Yi Li, Guixing Jin, Yutang Zhang, Wei Liang, Yunchun Chen, Changyin Zhao, Haiyan Li, Yiping Chen, Shenxun Shi, Kenneth S. Kendler, Jonathan Flint, Xumei Wang

**Affiliations:** a ShengJing Hospital of China Medical University, No.36 Sanhao Street, Heping District, Shenyang, Liaoning, 110817, PR China; b Wellcome Trust Centre for Human Genetics, Oxford OX3 7BN, UK; c Fudan University affiliated Huashan Hospital, No. 12 Wulumuqi Zhong Road, Shanghai, 200040, PR China; d Shanghai Jiao Tong University School of Medicine affiliated Shanghai Mental Health Centre, No. 600 Wan Ping Nan Road, Shanghai 200030, PR China; e Shanghai Tongji University affiliated Tongji Hospital, No. 389 Xinchun Road, Shanghai 200065, PR China; f Nanjing Brain Hospital, No. 264 Guangzhou Road, Nanjing, Jiangsu, 210029, PR China; g No. 4 Affiliated Hospital of Jiangsu University, No. 246 Nan Men Da Street, Zhenjiang, Jiangsu, 212001, PR China; h Zhejiang Traditional Chinese Medical Hospital, No. 54 You Dian Road, Hangzhou, Zhejiang 310006, PR China; i Tianjin Anding Hospital, No.13 Liu Lin Road, Hexi District, Tianjin, 300222, PR China; j Shandong Mental Health Center, No. 49 East Wenhua Road, Jinan, Shandong 250014, PR China; k No. 1 Hospital of Medical College of Xian Jiaotong University, No. 277 West Yan Ta Road, Xi'an, Shaanxi, 710061, PR China; l No.1 Hospital of Zhengzhou University, No.1 East Jianshe Road, Zhengzhou, Henan 450052, PR China; m No. 1 Mental Health Center Affiliated Harbin Medical University, No 23 You Zheng Jie, Nangang District, Harbin, Heilongjiang, PR China; n Mental Health Center of West China Hospital of Sichuan University, No. 28 Dian Xin Nan Jie, Wu Hou District, Chengdu, Sichuan 610041, PR China; o Beijing Anding Hospital, Capital Medical University, No.5 An Kang Hutong Deshengmen wai, Xicheng District, Beijing 100088, PR China; p Hebei Mental Health Center, No.572 Dongfeng Road, Baoding, Hebei 071000, PR China; q Shenzhen Kangning Hospital, No.1080, Cui Zu Street, Luo Hu, Shenzhen, 518020, PR China; r No. 3 Affiliated Hospital of Sun Yat-sen University, No.600 Tian He Road, Tian He District, Guangzhou, Guangdong, 510630, PR China; s No.1 Hospital of Shanxi Medical University, No. 85 Jiefang South Road, Taiyuan, Shanxi, 030001, PR China; t Mental Hospital of Jiangxi Province, No. 43 Shangfang Road, Nanchang, Jiangxi, 330029, PR China; u The First Affiliated Hospital of Jinan University, No.613 West Huangpu Avenue, Guangzhou, 510630, PR China; v Wuhan Mental Health Center, No.70, You Yi Road, Wuhan, 430022, PR China; w No.3 Hospital of Heilongjiang Province, No.135 Jiao Tong Lu, Beian, Heilongjiang, PR China; x Jilin Brain Hospital, No.98 Zhong Yang Xi Lu, Siping, Jilin, 136000, PR China; y The First Hospital of China Medical University, No.155 Nanjing Bei Jie, He Ping District, Shenyang, 110001, PR China; z Dalian No. 7 People's Hospital & Dalian Mental Health Center, No.179 Ling Shui Lu, Gan Jing Zi District, Dalian, PR China; aa The First Hospital of Hebei Medical University, No. 89 Donggang Road, Shijiazhuang, 050031, PR China; ab Lanzhou University Second Hospital, Second Clinical Medical College of Lanzhou University, No. 82, Cui Ying Men, Lanzhou, Gansu, 730030, PR China; ac Psychiatric Hospital of Henan Province, No.388 Jian She Zhong Lu, Xinxiang, Henan, PR China; ad The Fourth Military Medical University affiliated Xijing Hospital, No.17, Changle West Road, Xi'an, Shaanxi, 710032, PR China; ae No. 4 People's Hospital of Liaocheng, No. 47 Hua Yuan Bei Road, Liaocheng, Shandong, 252000, PR China; af Guangzhou Brain Hospital/Guangzhou Psychiatric Hospital, No.36 Ming Xin Lu, Fang Cun Da Dao, Li Wan District, Guangzhou 510370, PR China; ag Clinical Trial Service Unit, Richard Doll Building, Old Road Campus, Roosevelt Drive, Oxford, OX3 7LF, UK; ah Virginia Commonwealth University, Department of Psychiatry, Virginia Institute for Psychiatric and Behavioral Genetics, Richmond, VA 23298-0126, USA

**Keywords:** Major depressive disorder, Anxiety disorders, Neuroticism

## Abstract

**Objective:**

The personality trait of neuroticism is a risk factor for major depressive disorder (MDD), but this relationship has not been demonstrated in clinical samples from Asia.

**Methods:**

We examined a large-scale clinical study of Chinese Han women with recurrent major depression and community-acquired controls.

**Results:**

Elevated levels of neuroticism increased the risk for lifetime MDD (with an odds ratio of 1.37 per SD), contributed to the comorbidity of MDD with anxiety disorders, and predicted the onset and severity of MDD. Our findings largely replicate those obtained in clinical populations in Europe and US but differ in two ways: we did not find a relationship between melancholia and neuroticism; we found lower mean scores for neuroticism (3.6 in our community control sample).

**Limitations:**

Our findings do not apply to MDD in community-acquired samples and may be limited to Han Chinese women. It is not possible to determine whether the association between neuroticism and MDD reflects a causal relationship.

**Conclusions:**

Neuroticism acts as a risk factor for MDD in Chinese women, as it does in the West and may particularly predispose to comorbidity with anxiety disorders. Cultural factors may have an important effect on its measurement.

## 1 Introduction

Neuroticism is a longitudinally and culturally robust measure of emotional stability that emerges as a key dimension in almost all personality systems since proposed by Eysenck (Eysenck and Eysenck, 1975). The various manifestations of neuroticism in different assessments have been shown to exhibit considerable overlap (Aluja et al., 2002; McCrae et al., 1985; Zuckerman et al., 1993) and the trait is quite stable over many years (Wray et al., 2007). Neuroticism can be detected within a variety of different social strata and cultures (Eaves et al., 1989) and may even be recognized in the behavior of other, less complex, organisms (Gosling, 2001; Gosling et al., 2003).

Neuroticism is important in the study of human psychiatric disease because of evidence that it reflects predisposition to experiencing symptoms of anxiety and depression. High neuroticism scores are robustly associated with an increased risk for depression (Angst and Clayton, 1986; Hirschfeld et al., 1989; Kendell and DiScipio, 1968; Kendler et al., 1993; Wetzel et al., 1980), and experience of a depressive episode yields an elevation in neuroticism which persists after post-recovery (i.e., a scar effect) (Reich et al., 1987). Neuroticism is known to be a strong risk factor for the lifetime prevalence of major depressive disorder (MDD) (Alnaes and Torgersen, 1997; Hirschfeld et al., 1989; Roberts and Kendler, 1999; Rush et al., 1995).

Work in European and US populations indicates that the association between MDD and neuroticism is in part mediated by a common genetic susceptibility (Fanous et al., 2002) which also contributes to the correlation between MDD and anxiety disorders (Hettema et al., 2004; Hettema et al., 2006; Jardine et al., 1984; Kendler et al., 2007). While evidence indicates that the features of neuroticism are similar in different cultures and countries (McCrae and Terracciano, 2005), it is not clear whether the relation between neuroticism and MDD is present in non-Western populations.

In this paper, we report the results from a large-scale clinical study of Chinese Han women with recurrent MDD and matched controls. We aimed to evaluate three hypotheses: (1) neuroticism is related strongly to lifetime prevalence of MDD; (2) neuroticism increases the likelihood of comorbid disorders; (3) neuroticism is associated with the age at onset, number and maximal duration of episodes, and the pattern of reported symptoms.

## 2 Method

### 2.1 Subjects

Data for the present study draws upon the ongoing China, Oxford and VCU Experimental Research on Genetic Epidemiology (CONVERGE) study of MDD. Analyses were based on a total of 1970 cases recruited from 53 provincial mental health centers and psychiatric departments of general medical hospitals in 41 cities in 19 provinces and four central cities: Beijng, Shanghai, Tianjin and Chongqing and 2597 controls who were recruited from patients undergoing minor surgical procedures at general hospitals or from local community centers. All cases and controls were female and had four Han Chinese grandparents. Cases and controls were excluded if they had a pre-existing history of bipolar disorder, any type of psychosis or mental retardation. Cases were aged between 30 and 60, had two or more episodes of MDD, with the first episode occurring between 14 and 50 and had not abused drug or alcohol before the first episode of MDD. Controls were chosen to match the region of origin of cases, were aged between 40 and 60, had never experienced an episode of MDD and were not blood relatives of cases. An older minimal age of controls was used to reduce the chances that they might have a subsequent first onset of MDD. The mean age (and SD) of cases and controls in the dataset was respectively 45.1 (8.8) and 47.7 (5.5).

All subjects were interviewed using a computerized assessment system, which lasted on average 2 h for a case and 1 h for a control. All interviewers were trained by the CONVERGE team for a minimum of one week in the use of the interview. The interview includes assessment of psychopathology, demographic and personal characteristics, and psychosocial functioning. Interviews were tape-recorded and a proportion of them were listened to by the trained editors who provided feedback on the quality of the interviews.

The study protocol was approved centrally by the Ethical Review Board of Oxford University and the ethics committee in participating hospitals in China.

### 2.2 Measures

The diagnoses of depressive (dysthymia and MDD) and anxiety disorders (generalized anxiety disorder, panic disorder with or without agoraphobia) were established with the Composite International Diagnostic Interview (CIDI) (WHO lifetime version 2.1; Chinese version), which classifies diagnoses according to the Diagnostic and Statistical Manual of Mental Disorders (DSM-IV) criteria (American Psychiatric Association, 1994). The interview was originally translated into Mandarin by a team of psychiatrists in Psychiatry department of Huashan Hospital of Fudan University, Shanghai Mental Health Center of Jiao Tong University, School of Medicine and Psychiatry department of Tongji Hospital of Tongji University Shanghai Mental Health Centre with the translation reviewed and modified by members of the CONVERGE team. Phobias, divided into five subtypes (animal, situational, social and blood-injury, and agoraphobia) were diagnosed using an adaptation of DSM-III criteria requiring one or more unreasonable fears, including fears of different animals, social phobia and agoraphobia that objectively interfered with the respondent's life. The section on the assessment of phobias was translated by the CONVERGE team from the interview used in the Virginia Adult Twin Study of Psychiatric and Substance Use Disorders (VATSPUD) (Kendler and Prescott, 2006).

Additional information using instruments employed from VATSPSUD, translated and reviewed for accuracy by members of the CONVERGE team, was collected on neuroticism.

Neuroticism was measured with the 23-item Eysenck Personality Questionnaire(Eysenck and Eysenck, 1975), which was also an established instrument for measuring neuroticism.

Both the case and control interview were fully computerized into a bilingual system of Mandarin and English developed in house in Oxford, and called SysQ. Skip patterns were built into SysQ. Interviews were administered by trained interviewers and entered offline in real time onto SysQ, which was installed in the laptops. Once an interview was completed, a backup file containing all the previously entered interview data could be generated with database compatible format. The backup files together with an audio recording of the entire interview were uploaded to a designated server currently maintained in Beijing by a service provider. All the uploaded files in the Beijing server were then transferred to an Oxford server quarterly.

### 2.3 Statistical analysis

We performed logistic regression analyses to estimate the association of neuroticism with MDD and comorbid disorders (dysthymia, generalized anxiety disorder, panic disorder, agoraphobia,social phobia, animal phobia, situational phobia, and blood-injury phobia).MDD and comorbid disorders were coded as binary (1 = present, 0 = absent). Analyses of the relationship between neuroticism and the risk for lifetime MDD and comorbid disorders were conducted by binary logistic regression using SPSS version 17.0. Neuroticism scores were standardized so that the odds ratio (OR) reflected the alteration in the risk of MDD with every increase of 1 SD in neuroticism.

## 3 Results

**Fig. 1:**
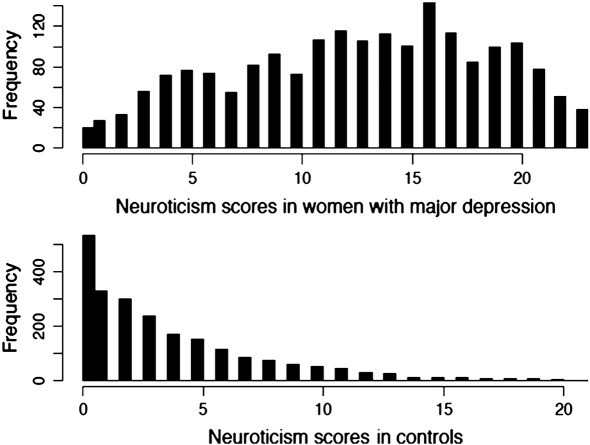
Distribution of neuroticism score in Chinese women with MDD and matched controls.

We obtained neuroticism scores on 1915 women with recurrent MDD and 2275 controls. The distribution of neuroticism scores is strikingly different in the two groups, “J” shaped in the controls and approximating a normal distribution in the cases (Fig. 1). The mean neuroticism score for cases is 12.7 (median 13) and the mean neuroticism score for controls is 3.6 (median 2). The difference between the two distributions is highly significant (t > 58, permutation derived P value < 0.0001).

Table 1 shows the odds ratios from the logistic regression analyses for MDD and comorbid disorders. In our full case–control sample, we found that increasing the neuroticism score by one standard deviation (s.d.) carries an odds ratio for MDD of 1.37. In our case sample, we also found that higher neuroticism scores significantly increased the risk of all comorbid disorders examined. For each s.d. increase in neuroticism, the highest risk increase was for generalized anxiety disorder (OR = 1.13) and the lowest was for phobia (OR = 1.06).

When we examined cases of MDD we found that higher neuroticism scores significantly increased the risk for the number of comorbid disorders (OR = 1.13), the number of MDD episodes (OR = 1.07), and the number of depressive symptoms (OR = 1.08) (Table 2). Using linear regression, we found a highly significant relationship between neuroticism and the age at onset of MDD (P value < 1 × 10^− 14^); neuroticism explained 3% of the variance in age of onset. Neuroticism scores were also modestly related to the maximal duration of episodes (P value = 0.004) explaining ~ 0.5% of the variance.

## 4 Discussion

We have examined the relationship between the personality trait of neuroticism and MDD in a large clinically ascertained sample of Chinese female patients with recurrent MDD. Our study has three major findings. First, neuroticism scores were strongly related to the lifetime prevalence of MDD. Second, an increase in neuroticism scores increases the likelihood of comorbid disorders in those with MDD. Third, in those affected with MDD, neuroticism scores are associated with several important clinical features of MDD. Higher scores predicted a younger age of onset, more episodes of depression, a higher symptom score and a longer maximal duration of an MDD episode. These findings establish neuroticism as an important predictor of the onset and severity of MDD in our sample.

To our knowledge this is the first study of neuroticism in Chinese female patients with MDD. Previous work among adolescents and University students in China has shown a relationship between personality features and depressive symptoms (Lu, 1994; Song et al., 2008; Wang et al., 2010), but no one has documented the effect of neuroticism on the clinical features of MDD.

Our findings replicate the results obtained in clinical populations in Europe and US in a number of ways. We find that the effect of neuroticism on MDD is of a similar magnitude to that reported elsewhere in the world. Typically Western studies estimate the OR of neuroticism to be about 1.5, where the OR reflects the alteration in risk of MDD with each increase of 1 standard deviation in the neuroticism score (Kendler et al., 2006). Our estimate is only slightly less: 1.37. In Western studies, neuroticism has been shown to account for about a third of the comorbidity with internalizing disorders (which include anxiety and depression) (Khan et al., 2005) and to correlate with the severity of depression (indexed by the duration and number of episodes) (Alnaes and Torgersen, 1997; Duggan et al., 1990; Scott et al., 1995). We find that in Chinese patients high neuroticism scores also predispose to higher comorbidity and scores correlate with disease severity. Previous research found that neuroticism accounted for a significant part of the lifetime comorbidity of common psychiatric disorders (internalizing disorders and externalizing disorders) and suggests neuroticism as a potential general underlying vulnerability factor for psychopathology (Clark et al., 1994; Khan et al., 2005; Krueger and Markon, 2001; Sher and Trull, 1994). As a cross sectional analysis, our results showed that elevated neuroticism scores were associated with lifetime comorbidity.

Neuroticism scores in our sample differ from those reported in the West: most strikingly, the mean score is much lower. Studies in European community acquired populations report mean neuroticism scores with a distribution approximating normality centered around a mean of 10 (Eaves et al., 1989; Martin et al., 2000); we find a mean of 3.6 in the community acquired sample of Chinese women, and a highly skewed distribution. The apparent lower levels of neuroticism in our sample are potentially important, as they may be related to the lower rates of MDD in China compared to the West: the 12-month prevalence of MD is approximately 7% in the US (Kessler et al., 2003) and 2% in China (Phillips et al., 2009).

Personality factors, including neuroticism, are known to vary across cultures, and there are varying, sometimes conflicting, explanations offered (Allik and McCrae, 2004; Barrett and Eysenck, 1984; Hofstede and McCrae, 2004; Lynn and Martin, 1995). However explaining cross-cultural variation with respect to China is particularly difficult because there has been relatively little research into personality (psychology was only reinstated as an approved discipline in the PRC in the 1980s).

Two points deserve note. First personality factors in China may not be identical to those observed in the West. While the majority of work since the 1980s has relied on measures developed in Western countries (Cheung, 2004; Zhang, 1988), since the development and use of indigenous personality inventories, such as the Chinese Personality Assessment Inventory (Cheung et al., 1996), it has been argued that a different personality model (six-factor model) is superior to the model commonly found in the West (five factor model) (Cheung et al., 2001). Second, cultural variation in neuroticism scores may be due to methodological differences between studies (Geisinger, 1994; van de Vijver and Leung, 1997). For example the meaning of an item may alter subtly after translation, and the context in which the test is administered may bias responses, a potentially important factor in our work. Even though subjects fill in the questionnaire themselves, it is likely that their responses are affected by the respect Chinese traditionally hold for people in authority, such as doctors. However this factor would not explain the difference in neuroticism scores seen between patients and controls.

The results of our study should be interpreted in the context of three potential methodological limitations. First, all cases in our study were selected from a clinical sample. Our results may not apply to MDD in community-acquired samples. Second, the sample was limited to Chinese Han women, so the results may not generalize to men and to individuals from other ethnic groups. Third, we cannot, in this sample, gain direct insight into the causal relationship between neuroticism and MDD as we assessed in a single interview the level of neuroticism and the lifetime history of MDD.

The results that we observe probably reflect several mechanisms. Most important is probably the fact that neuroticism reflects an underlying liability to MDD (Boyce et al., 1991; Fanous et al., 2007; Kendler et al., 1993; Nystrom and Lindegrard, 1975). At least two other mechanisms likely play a role: a direct effect of MDD on the level of neuroticism for those currently in an episode (“state” effect) (Coppen and Metcalfe, 1965; Fanous et al., 2007; Farmer et al., 2002; Hirschfeld et al., 1983; Kendler et al., 1993; Kerr et al., 1970) and a long-term effect of prior depressive episodes on neuroticism (“scar” effect) (Fanous et al., 2007; Farmer et al., 2002; Kendler et al., 1993; Reich et al., 1987).

## Role of funding source

Funding for this study was provided by the Wellcome Trust; the Wellcome Trust had no further role in study design; in the collection, analysis and interpretation of data; in the writing of the report; and in the decision to submit the paper for publication.

## Conflict of interest

All authors declare they have no conflicts of interest including any financial, personal or other relationships with other people or organizations within three years of beginning the work submitted that could inappropriately influence, or be perceived to influence, their work.

## Figures and Tables

**Table 1:** Neuroticism as a predictor for MDD and comorbid disorders.

	OR	95%CI	p-value
MDD	1.37	1.35 to 1.40	2.2 × 10^− 263^
MD + GAD	1.13	1.11 to 1.15	3.4 × 10^− 34^
MD + panic	1.09	1.06 to 1.12	3.1 × 10^− 9^
MD + dysthymia	1.12	1.10 to 1.15	8.2 × 10^− 22^
MD + phobia	1.06	1.04 to 1.08	4.2 × 10^− 11^
MD + agoraphobia	1.08	1.06 to 1.10	8.3 × 10^− 17^
MD + social	1.07	1.05 to 1.09	4.8 × 10^− 14^
MD + Animal	1.03	1.02 to 1.05	4.0 × 10^− 5^
MD + situational	1.06	1.04 to 1.07	1.4 × 10^− 10^
MD + blood	1.05	1.04 to 1.07	7.9 × 10^− 10^

Odds ratios (OR) with 95% confidence intervals (95% CI) and their statistical significance (p-value) are reported. An odds ratio of > 1 represents the increase in risk of MDD and comorbidity associated with each standard deviation (sd) increase in the score of the neuroticism. An odds ratio of < 1 represents the decrease in risk of MDD and comorbidity associated with each sd increase in neuroticism score.

**Table 2:** Neuroticism as predictor of the number of comorbid disorders, the pattern of reported depressive symptoms, and number of episodes of MDD.

	OR	95%CI	p-value
Number of comorbid diseases	1.13	1.11 to 1.15	2.4 × 10^− 55^
Number of MDD symptoms	1.08	1.07 to 1.10	1.3 × 10^− 25^
Number of melancholia	1.00	0.99 to 1.02	0.676
Number of MDD episodes	1.07	1.04 to 1.10	5.4 × 10^− 6^

Odds ratios (OR) with 95% confidence intervals (95%CI) and their statistical significance (p-value) are reported. An odds ratio of > 1 represents the increase in risk of MDD and comorbidity associated with each standard deviation increase in the score of the neuroticism. An odds ratio of < 1 represents the decrease in risk of MDD and comorbidity associated with each standard deviation increase in neuroticism score.
